# The association between social determinants, lifestyle and metabolic factors and the onset of secondary glenohumeral joint osteoarthritis: a cohort study of adults in the UK

**DOI:** 10.3389/fpubh.2025.1718963

**Published:** 2026-01-27

**Authors:** Yifeng Yan, Jiabo Yuan, Jie Mei, Dapeng Zhang, Yao Liu, Yong Sun, Jianing Liu, Qiang He

**Affiliations:** 1 Department of Orthopedics, The Second Affiliated Hospital of Nanjing University of Chinese Medicine, Nanjing, Jiangsu, China; 2 Graduate School, Heilongjiang University of Chinese Medicine, Haerbin, Heilongjiang, China; 3 Department of Ophthalmology, The Second Hospital Affiliated to Shandong University of Traditional Chinese Medicine, Jinan, Shandong, China; 4 Department of Orthopedics, Nanjing Hospital of Chinese Medicine Affiliated to Nanjing University of Chinese Medicine, Nanjing, Jiangsu, China; 5 Shandong Cancer Hospital and Institute, Shandong First Medical University and Shandong Academy of Medical Sciences, Jinan, Shandong, China; 6 Shandong Provincial Hospital, Jinan, Shandong, China; 7 Department of Central Laboratory, Shandong Provincial Hospital Affiliated to Shandong First Medical University, Jinan, Shandong, China; 8 Department of Orthopedics, Shandong Provincial Hospital Affiliated to Shandong First Medical University, Jinan, Shandong, China

**Keywords:** incidence risk, lifestyle, metabolic, secondary glenohumeral joint osteoarthritis, social determinants

## Abstract

**Objective:**

Secondary Glenohumeral Joint Osteoarthritis (GJO) is a degenerative condition of non-weight-bearing joints. While age and gender are known risk factors, the role of modifiable factors remains underexplored. This study aimed to assess the association of social determinants, lifestyle, and metabolic factors with the incidence of secondary GJO.

**Methods:**

We included 26,708 UK Biobank participants without OA at baseline and who reported secondary GJO only at follow-up. Median follow-up duration was 8.85 years. A Social Determinants, Lifestyle, and Metabolic (SLM) score was developed based on 17 variables: 4 social, 5 lifestyle, and 8 metabolic indicators. Each adverse factor contributed one point (range: 0–17). Cox proportional hazards models were used to evaluate the relationship between baseline SLM score and incident secondary GJO. Subgroup analyses were stratified by age and gender.

**Results:**

There was a significant linear trend between SLM score and secondary GJO risk (P < 0.001). Participants with higher SLM scores had a 3.75-fold increased risk of developing secondary GJO (AHR = 3.75, 95% CI: 1.87–7.51, *P* = 0.0002). Higher social determinant scores (AHR = 3.35, P = 6.8 × 10^−6^) and metabolic scores (AHR = 1.62, *P* = 0.045) were independently associated with increased risk, while lifestyle factors showed a nonsignificant trend. Subgroup analyses revealed stronger associations in women, men, and individuals under 60 years old.

**Conclusions:**

Modifiable social and metabolic factors significantly influence the risk of secondary GJO. Early identification and intervention targeting these factors may aid in the prevention of this condition.

## 1 Introduction

With the acceleration of global population aging, the burden of musculoskeletal system diseases is increasing, especially non-communicable chronic joint disorders, which have a significant impact on the functional impairment and quality of life of the older adult. Glenohumeral joint osteoarthritis (GJO) is one of the common musculoskeletal diseases in clinical practice, seriously affecting the upper limb function and quality of life of patients (1). It is reported that 5% to 21% of American adults have shoulder pain, and nearly one-third of people over 60 years old worldwide suffer from GJO (2, 3). Among them, secondary GJO usually occurs after trauma, chronic strain, joint instability or systemic diseases, and has an earlier onset, more complex causes and a more rapid progression than primary GJO (4). According to a relevant prediction published in 2010, the number of shoulder arthroplasties is expected to increase by 192% to 322% from 2007 to 2015, while the proportion of revision surgeries is expected to rise from about 4.5% to 7.0% (5), reflecting the treatment challenges of secondary GJO in orthopedic clinical practice. Due to the fact that existing treatment methods mainly focus on symptom relief and functional improvement, there is still a lack of clear primary prevention strategies. Therefore, identifying the potential modifiable risk factors of secondary GJO will provide an important basis for formulating effective public health prevention and intervention measures.

Unlike knee and hip OA, which directly cause disability, secondary GJO causes upper limb dysfunction, significantly limiting patients' daily activities and work ability and seriously affecting their quality of life (6). In addition, the etiology of secondary GJO is complex, often resulting from the combined effects of previous trauma, occupation-related strain and systemic diseases, making its pathogenesis more diverse than that of other joint OA (7). Previous studies have mostly focused on unchangeable risk factors such as age and gender (8–10), while paying insufficient attention to modifiable factors such as social determinants, lifestyle behaviors and metabolic abnormalities. However, in recent years, many studies have suggested that low socioeconomic status, mental and psychological stress, unhealthy lifestyles (such as smoking, unbalanced diet, lack of exercise) and metabolic abnormal states (such as obesity, hypertension, dyslipidemia, etc.) may participate in the degeneration of cartilage and joint structure through mechanisms such as chronic inflammatory response, oxidative stress damage and immune imbalance, thereby playing an important role in the occurrence and development of secondary GJO (3, 11–15). These results suggest that the risk of secondary GJO is not completely uncontrollable, but may be significantly influenced by modifiable environmental and behavioral factors.

The “Social Determinants of Health” framework proposed by the World Health Organization clearly states that multiple dimensions such as social structural factors, lifestyle, and metabolic status should be incorporated into the prevention and control system of chronic diseases, and high-risk groups should be identified and intervened through cross-level integration (16). Although this theory has been widely applied in various chronic diseases, there is still a lack of systematic research on the combined impact of these three types of factors in the field of Secondary GJO, especially high-quality epidemiological evidence from large sample, long-term follow-up prospective cohorts.

In view of this, to explore whether and how social determinants, lifestyle, and metabolic factors affect the incidence of Secondary GJO, this study utilized the UK Biobank, a large prospective cohort, to construct a comprehensive score integrating social determinants, lifestyle, and metabolic status (SLM score). The aim is to systematically assess the relationship between the SLM score and the incidence of Secondary GJO, provide a simple and effective tool for identifying high-risk groups in clinical practice, and offer evidence-based support for the formulation of prevention and precise intervention strategies for Secondary GJO.

## 2 Materials and methods

### 2.1 Data source and study population

This is a prospective cohort study based on the data from the UK Biobank (the study protocol is available online: www.ukbiobank.ac.uk) (17). The UK Biobank is a large prospective cohort study covering 520,000 adults aged 39 to 73 years in the UK. Participants came from one of 22 assessment centers in England, Scotland, and Wales, where they completed touchscreen and nurse-led questionnaires, underwent physical measurements, and provided biological samples.

**Figure 1:**
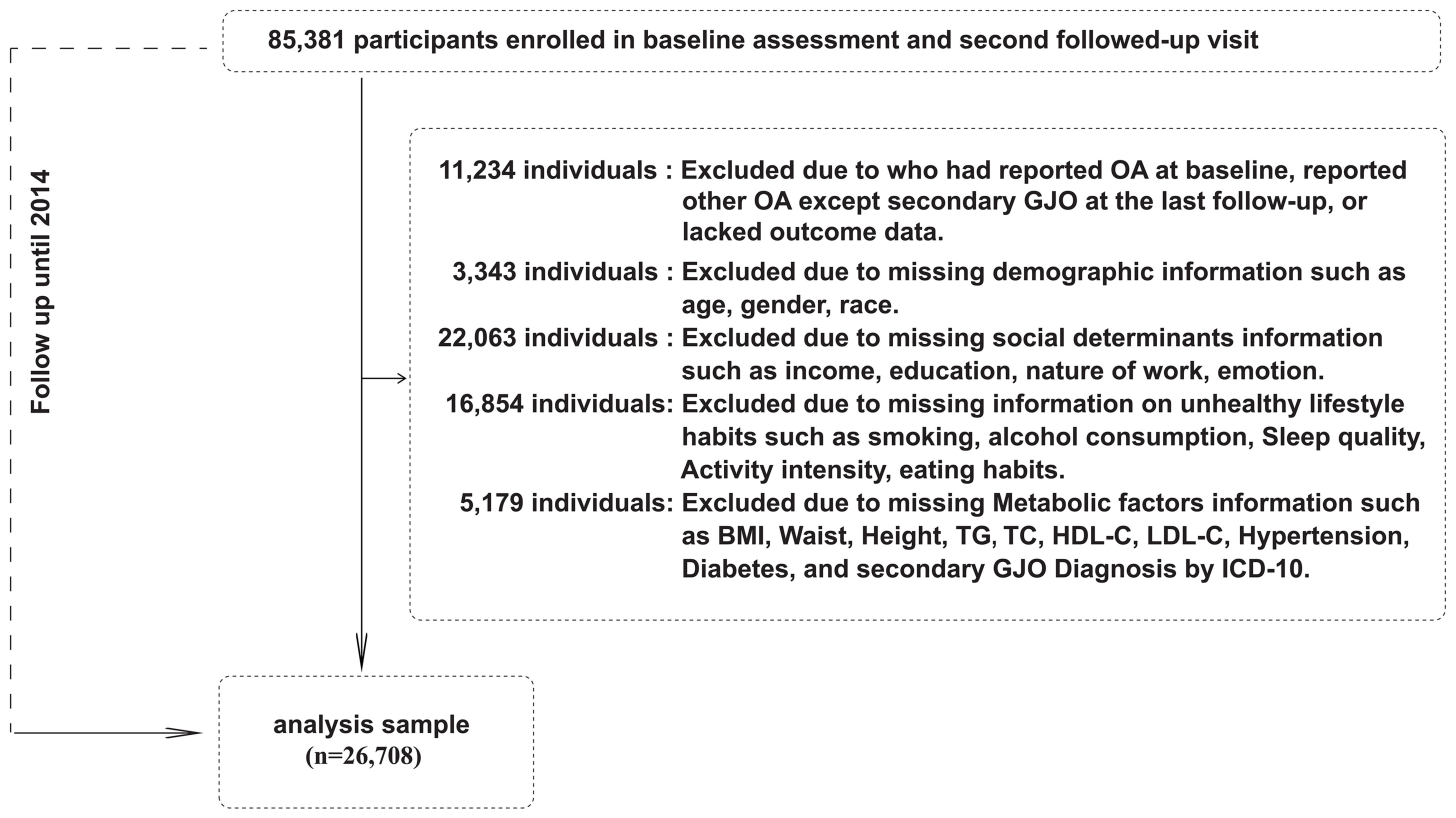
Flowchart of participant selection.

This study included adults (*n* = 26,708) from the UK Biobank who completed the baseline assessment between 2006 and 2010 and had relevant follow-up data from the second follow-up in 2014. The primary exposure was the Social determinant, lifestyle and metabolic factor scores (SLM scores) measured at baseline (2006–2010), and the outcome was incident secondary GJO identified by 2014. Incident secondary GJO was ascertained based on linked hospital admission records (Hospital Episode Statistics) using ICD-10 diagnostic codes. The median follow-up time was 8.85 years (interquartile range: 7.15 to 10.75 years). All participants provided written informed consent, and the UK Biobank was conducted with ethical approval from the Northwest Multi-Centre Research Ethics Committee (www.ukbiobank.ac.uk/ethics/). The detailed participant selection process is illustrated in Figure 1.

### 2.2 Construction of social determinant, lifestyle and metabolic factor scores and definition of outcomes

**(1) SLM scores**: In this study, we collected and evaluated 17 baseline social and personal modifiable risk factors. The 17 risk factors were classified into 4 social determinants (household income below the national average, low education level, engaging in heavy physical labor, individual-level data such as seeking medical treatment for personal anxiety, tension or depression), 5 lifestyle factors (current smoking status, current drinking status, unhealthy diet, low physical activity and poor sleep quality), and 8 metabolic risk factors (obesity, abdominal obesity, abnormal triglycerides, abnormal total cholesterol, abnormal HDL, abnormal LDL, hypertension, and diabetes). We assigned a score of 1 for adverse risk factors and 0 for good habits. The SLM scores range from 0 to 17. The detailed definitions and thresholds for each component are provided in Supplementary Table S1. In alignment with prior literature and to enhance interpretability, we divided the total SLM score into three categories based on tertiles of the distribution in the total cohort: Low (score: 0–4) Middle (score: 5–8), High (score: 9–17). This categorization ensured a balanced distribution of participants across groups and allowed for subgroup risk stratification in the analysis. Poor mental state is considered a multi-faceted structure reflecting social factors and has been classified as a social determinant in many studies (18, 19). For dietary habits, we used the simplified Mediterranean diet score (aMED simplified version), and the detailed content of the aMED simplified version score can be found in Supplementary Table S2.

**(2) Diagnosis of secondary GJO:** Secondary GJO was identified from hospital inpatient records using ICD-10 codes (M19.11 and M19.21), while primary GJO (M19.01) and unspecified GJO (M19.81, M19.91) were excluded to minimize misclassification. Detailed ICD-10 codes are provided in Supplementary Table S3.

### 2.3 Covariates

The covariates included in the study were basic demographic factors, age [continuous variable, divided into two groups based on common epidemiological thresholds: young (< 60 years old) and older adult (≥ 60 years old)], gender (male, female), and race as self-identified by the participants (White Lineage, Asian Lineage, Mixed or Other Lineage, Black Lineage); social determinants, income (Low, Middle, High), education level (Below College or University degree, College or University degree), occupation (involving heavy manual or physical labor, classified as Never/rarely/Sometimes, Usually, Always), personal medical history of neurosis, anxiety, tension or depression (Yes, No); lifestyle factors, smoking status (Never, Former or Current smoker), alcohol consumption (Never, Frequent Drinker, Occasional Drinker), diet (aMED simplified version: medium/high score, low score), meeting international recommended exercise intensity (Yes, No), sleep quality (poor, good); metabolic-related factors, BMI (continuous, normal, overweight and obese), WHtR (continuous, normal, pre-central obesity and severe central obesity), hypertension (Yes, No), dyslipidemia (Yes, No), hyperglycemia (Yes, No). Hypertension was defined as systolic blood pressure greater than 140 mmHg or diastolic blood pressure greater than 90 mmHg or ICD-10 diagnosis; hyperglycemia was defined as blood glucose greater than 5.6 mmol/L (including prediabetes and diabetes) or ICD-10 diagnosis; The definition of dyslipidemia refers to the American NCEP ATP III (Adult Treatment Panel III) standard or ICD-10 diagnosis. Due to the strong association between Secondary GJO and age and gender (8–10), the study further divided the population into male, female, young group (< 60 years old) and older adult group (≥60 years old) for subsequent subgroup analysis.

### 2.4 Statistical analysis

Descriptive statistics were used to describe the UKB cohort study population from 2006 to 2014 (Table 1) to examine the differences in characteristics such as age, gender, social determinants, lifestyle, and metabolic factors among participants with Primary GJO and Secondary GJO. For categorical variables, frequencies and percentages were used and compared using the chi-square test. For continuous variables, the median (interquartile range [IQR]) was used and compared using the Kruskal-Wallis *H* test. The relationship between the SLM score at baseline and the risk of Secondary GJO at the end of follow-up was evaluated using Cox proportional hazards regression models, and three models were constructed: Model 1 (crude model): no adjustment for any covariates; Model 2: adjusted for age; Model 3 (fully adjusted model): further adjusted for gender and ethnicity on the basis of Model 2. Further subgroup analyses were stratified by participants' age and gender to explore the heterogeneity of this association in different characteristic populations. Results were reported as hazard ratios (HRs) and their 95% confidence intervals (95% CIs). All statistical analyses were performed using R software version 4.3.1, and a two-sided *p* < 0.05 was considered statistically significant.

**Table 1:** Baseline characteristics of the study population.

**Variable**	**Overall (*n* = 26,708)**	**Primary GJO (*n* = 26,572)**	**Secondary GJO (*n* = 136)**	***P* value**
Age, median (IQR)	52.00 (47.00–57.00)	52.00 (47.00–57.00)	55.00 (49.00–58.00)	**0.003**
Female, *n* (%)	13,317 (49.86)	13,255 (49.88)	62 (45.59)	0.300
Race, *n* (%)				0.300
White Lineage	25,708 (96.26)	25,578 (96.26)	130 (95.59)	
Asian Lineage	419 (1.57)	417 (1.57)	2 (1.47)	
Mixed or Other Lineage	366 (1.37)	362 (1.36)	4 (2.94)	
Black Lineage	215 (0.80)	215 (0.81)	0 (0.00)	
**Social determinants**				
Income, *n* (%)				0.130
Low	3,216 (12.04)	3,193 (12.02)	23 (16.91)	
Middle	12,331 (46.17)	12,266 (46.16)	65 (47.79)	
High	11,161 (41.79)	11,113 (41.82)	48 (35.30)	
Education, *n* (%)				**<** **0.001**
Low levels of education	14,165 (53.04)	14,066 (52.94)	99 (72.79)	
High levels of education	12,543 (46.96)	12,506 (47.06)	37 (37.21)	
Nature of work (involves heavy manual or physical), n (%)				**0.018**
Never/rarely/Sometimes	19,216 (71.95)	19,133 (72.00)	83 (61.03)	
Usually	3,696 (13.84)	3,670 (13.81)	26 (19.12)	
Always	3,796 (14.21)	3,769 (14.19)	27 (19.85)	
Individuals seeking medical treatment for anxiety, tension or depression, *n* (%)	8,037 (30.09)	7,992 (30.08)	45 (33.09)	0.400
**Lifestyle risk factors**				
smoke, *n* (%)	1,745 (6.53)	1,736 (6.53)	9 (6.62)	>0.900
drink, *n* (%)	12,511 (46.84)	12,443 (46.83)	68 (50.00)	0.400
aMED, *n* (%)	18,654 (69.84)	18,552 (69.82)	102 (75.00)	0.200
Suggested Exercise Intensity, *n* (%)				**0.009**
No	4,872 (18.24)	4,859 (18.29)	13 (9.56)	
Yes	21,836 (81.76)	21,713 (81.71)	123 (90.44)	
Quality of sleep, *n* (%)				**0.016**
Good	20,583 (77.07)	20,490 (77.11)	93 (68.38)	
Poor	6,125 (22.93)	6,082 (22.89)	43 (31.62)	
**Metabolic factors**				
BMI (kg/m^2^), median (IQR)	26.20 (23.80–29.00)	26.20 (23.80–29.00)	27.40 (24.50–30.55)	**0.002**
WHtR, median (IQR)	0.51 (0.47–0.56)	0.51 (0.47–0.56)	0.54 (0.49–0.58)	**< 0.001**
TG (≥ 1.7 mmol/L), *n* (%)	9,460 (35.42)	9,402 (35.38)	58 (42.65)	0.077
TC(≥ 5.2 mmol/L), *n* (%)	17,848 (66.83)	17,751 (66.80)	97 (71.32)	0.300
LDL-C(≥ 3.4 mmol/L), *n* (%)	15,365 (57.53)	15,278 (57.50)	87 (63.97)	0.130
HDL-C(< 1.0 mmol/L), *n* (%)	10,892 (40.78)	10,844 (40.81)	48 (35.29)	0.200
Hypertension, *n* (%)	2,459 (9.21)	2,439 (9.18)	20 (14.71)	**0.026**
Diabetes, *n* (%)	1,263 (4.73)	1,257 (4.73)	6 (4.41)	0.900

Note: bold values to indicate “statistically significant differences”.

## 3 Results

### 3.1 Characteristics of the study population

We included 26,708 adult participants who did not report OA at baseline and excluded those who reported other types of OA except for Secondary GJO at the last follow-up. Among them, 136 individuals reported Secondary GJO after a median (IQR) follow-up of 8.85 (7.15 - 10.75) years. Table 1 presents the basic characteristics of the included population, with a median age of 52 years in the study population. Compared to the Primary GJO group, the Secondary GJO group was older, had a higher proportion of males; in terms of social determinants, the Secondary GJO group had a higher proportion of individuals with low education, low income, engaging in heavy physical labor, and seeking medical treatment for personal neurological, anxiety, tension or depression issues; in terms of lifestyle, the Secondary GJO group had a higher proportion of current drinkers, individuals with unhealthy diets, and those with poor sleep quality. Additionally, individuals in the Secondary GJO group performed worse in various metabolic factor indicators.

### 3.2 Significant association between baseline SLM score and the risk of Secondary GJO at the end of follow-up

**Figure 2:**
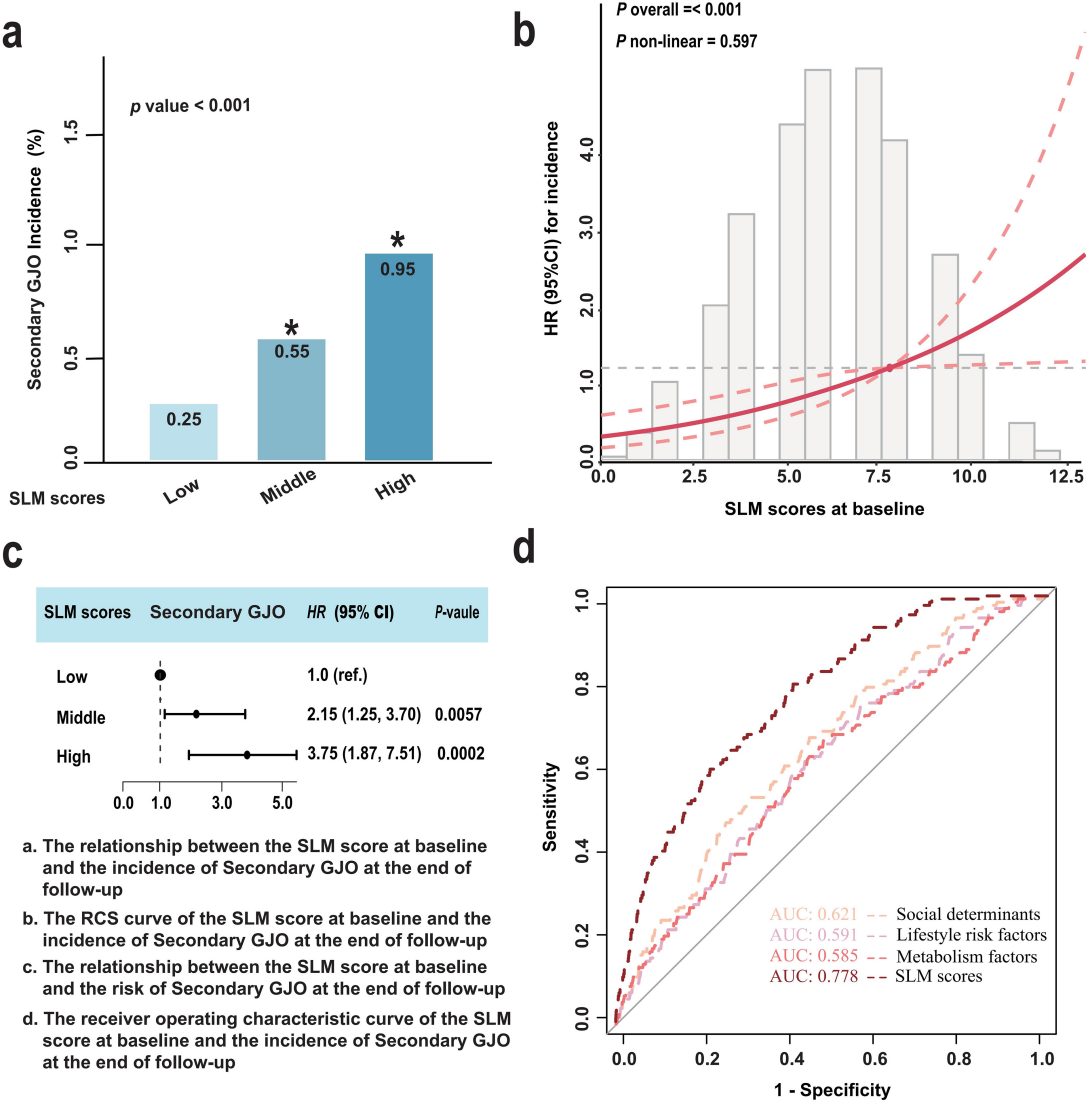
Association between baseline SLM scores and the incidence of secondary GJO during follow-up. **(a)** Incidence rates of secondary GJO across low, middle, and high categories of SLM scores at baseline. Higher SLM scores were associated with significantly increased incidence (*P* < 0.008). **(b)** Restricted cubic spline (RCS) plot showing a linear association between baseline SLM scores and Hand OA risk at follow-up (*P*-overall = < 0.001; *P*-nonlinear = 0.597). **(c)** Adjusted hazard ratios (HRs) with 95% confidence intervals (CIs) for secondary GJO by SLM score category. Compared with the low SLM score group, participants with high SLM scores had a significantly increased risk of secondary GJO (AHR = 3.75, 95% CI: 1.87–7.51, *P* = 0.0002). **(d)** Receiver operating characteristic (ROC) curves comparing the predictive ability of the SLM score vs. individual domains: social determinants, lifestyle factors, and metabolic factors. The area under the curve (AUC) for the SLM score was 0.778, outperforming the individual components.

Figure 2 shows the relationship between the baseline SLM score and the risk of Secondary GJO at the end of follow-up. Figure 2a indicates a significant association between a higher baseline SLM score and a higher incidence of Secondary GJO at the end of follow-up (*P* < 0.05). Figure 2b shows a significant linear association between the baseline SLM score and the risk of Secondary GJO at the end of follow-up (*P* < 0.001). Figure 2c shows that compared to a lower baseline SLM score, a higher SLM score increased the risk of Secondary GJO at the end of follow-up by 3.75 times (AHRs = 3.75, 95% CI: 1.87–7.51, *P* = 0.0002). Figure 2d shows that the AUC of the SLM score was 0.778, significantly higher than that of social determinants (0.621), lifestyle (0.591), and metabolic factors (0.585) individually, indicating that the multi-dimensional integrated model has better discriminatory ability in predicting Secondary GJO. (For more details, see Supplementary Tables S4, S5).

### 3.3 Relationship between baseline scores of social determinants, lifestyle, and metabolic factors and the risk of Secondary GJO at the end of follow-up

Table 2 shows the relationship between baseline scores of social determinants, lifestyle, and metabolic factors and the risk of Secondary GJO at the end of follow-up. Compared to a lower baseline score of social determinants, a higher score increased the risk of Secondary GJO at the end of follow-up by 3.35 times (AHRs = 3.35, 95% CI: 1.98 - 5.67, *P* = 6.8 × 10^−6^); compared to a lower baseline score of metabolic factors, a higher score increased the risk of Secondary GJO at the end of follow-up by 1.62 times (AHRs = 1.62, 95% CI: 1.01 - 2.59, *P* = 0.045); although the lifestyle score also showed a similar trend (AHRs = 1.60, 95% CI: 0.58 - 4.41, *P* = 0.368), no significant association was found (*P* > 0.05). As illustrated in Supplementary Figure S1, participants with higher baseline scores of social determinants and metabolic factors exhibited a progressively higher cumulative incidence of secondary GJO across score categories, whereas the trend for lifestyle factors was not statistically significant. Supplementary Figure S2 further demonstrated the dose–response relationships using restricted cubic spline models, showing a significant overall association for social determinants and metabolic factors with the risk of secondary GJO. The spline curves indicated a largely linear increasing pattern across the score range, while no clear nonlinear association was observed for lifestyle factors.

**Table 2:** The relationship between social determinants scores, lifestyle risk factors and metabolic factors scoresat baseline and the risk of Secondary GJO at the end of follow-up.

**Variable**	**Model 1**	***P* value**	**Model 2**	***P* value**	**Model 3**	***P* value**
**Social determinants (0-4, scores)**						
Low scores	Ref		Ref		ref	
Middle scores	1.56 (0.88–2.75)	0.126	1.55 (0.88–2.74)	0.001	1.59 (0.90–2.81)	0.109
High scores	3.26 (1.93–5.50)	**9.94e−06**	3.23 (1.91–5.45)	**1.20e−05**	3.35 (1.98–5.67)	**6.8e−06**
**Lifestyle risk factors (0–5, scores)**						
Low scores	Ref		Ref		ref	
Middle scores	1.25 (0.88–1.76)	0.216	1.27 (0.89–1.79)	0.184	1.27 (0.90–1.80)	0.176
High scores	1.54 (0.56–4.25)	0.406	1.59 (0.57–4.38)	0.373	1.60 (0.58–4.41)	0.368
**Metabolic factors (0–8, scores)**						
Low scores	Ref		Ref		ref	
Middle scores	1.52 (0.97–2.36)	0.066	1.48 (0.95–2.30)	0.087	1.47 (0.94–2.29)	0.091
High scores	1.69 (1.06–2.68)	**0.027**	1.64 (1.03–2.61)	**0.037**	1.62 (1.01–2.59)	**0.045**
Model 1: Adjust No						
Model 2: Adjust Age.						
Model 3: Adjust Age, Sex, Race.						

Note: bold values to indicate “statistically significant differences”.

### 3.4 Sensitivity analysis

Table 3 presents the subgroup analysis based on age and gender. In the overall population, compared with a lower SLM score at baseline, a higher SLM score was significantly associated with an increased risk of Secondary GJO at the end of follow-up in the following subgroups: adults under 60 years old (AHR = 3.65, 95% CI: 1.64 - 8.13, *P* = 0.001), females (AHR = 3.12, 95% CI: 1.13 - 8.63, *P* = 0.029), and males (AHR = 4.28, 95% CI: 1.63 - 11.25, *P* = 0.003). Although the above trend was also observed in adults over 60 years old (AHR = 3.34, 95% CI: 0.79 - 14.01, *P* = 0.099), no significant association was found (*P* > 0.05). Supplementary Figure S3 shows the RCS curves of the relationship between the SLM score at baseline and the incidence of Secondary GJO at the end of follow-up based on age and gender groups.

**Table 3:** Subgroup analysis based on age and gender.

**SLM (0–17, scores)**	**Model 1**	***P* value**	***P* for interaction**	**Model 2**	***P* value**	***P* for interaction**	**Model 3**	***P* value**	***P* for interaction**
**Subgroup (Age)**	0.812			0.830			0.824		
**Young (18-59, years)**									
Low scores	ref			ref			ref		
Middle scores	2.35 (1.28–4.30)	**0.006**		2.31 (1.26–4.24)	**0.007**		2.33 (1.27–4.27)	**0.006**	
High scores	3.67 (1.65–8.18)	**0.001**		3.63 (1.63–8.07)	**0.002**		3.65 (1.64–8.13)	**0.001**	
**Old (**>=**60, years)**									
Low scores	ref			ref			ref		
Middle scores	1.45 (0.43–4.87)	0.552		1.44 (0.43–4.86)	0.552		1.43 (0.43–4.82)	0.561	
High scores	3.46 (0.83–14.49)	0.089		3.38 (0.80–14.18)	0.096		3.34 (0.79–14.01)	0.099	
**Subgroup (Sex)**			0.687			0.711			0.713
**Sex (Female)**									
Low scores	ref			ref			ref		
Middle scores	2.25 (1.06–4.76)	**0.034**		2.16 (1.02–4.59)	**0.043**		2.17 (1.02–4.59)	**0.044**	
High scores	3.32 (1.20–9.15)	**0.020**		3.10 (1.12–8.58)	**0.029**		3.12 (1.13–8.63)	**0.029**	
**Sex (Male)**									
Low scores	ref			ref			ref		
Middle scores	2.11 (0.96–4.62)	0.063		2.09 (0.96–4.60)	0.064		2.13 (0.97–4.67)	0.059	
High scores	4.27 (1.62–11.21)	**0.003**		4.25 (1.62–11.16)	**0.003**		4.28 (1.63–11.25)	**0.003**	
Model 1: Adjust No									
Model 2: Adjust Age or Sex.									
Model 3: Adjust Age or Sex, Race.									

Note: bold values to indicate “statistically significant differences”.

## 4 Discussion

This study evaluated the relationship between three types of modifiable risk factors - social determinants, lifestyle, and metabolic factors - and the incidence of secondary GJO based on the UK Biobank system. By constructing an SLM scoring system consisting of 17 items, it was found that a higher SLM score was significantly associated with an increased risk of secondary GJO, and a dose-response relationship was observed. Subgroup analyses further revealed that the predictive value of the SLM score was present in both genders, and individuals under 60 years of age, suggesting that the SLM score not only has acceptable predictive performance but also possesses discriminative utility for identifying vulnerable subpopulations. Although the area under the curve (AUC) value of 0.778 reflects moderate discrimination, the strength of the SLM score lies in its utility for population-level risk stratification rather than individual-level clinical diagnosis. Its primary value is to help flag high-risk subgroups for targeted public health interventions and early prevention strategies, rather than serving as a standalone tool for definitive diagnostic decision-making in clinical settings.

Social determinants of health (SDOH) have long been neglected in OA research, but their role in the development of chronic diseases is increasingly recognized. This study found that a higher SDOH score was significantly associated with an increased risk of secondary GJO (AHRs = 3.35). This may be closely related to factors such as educational level, income level, and the nature of physical labor, which affect access to health resources, the formation of health awareness, and preventive behaviors for chronic diseases. In addition, mental and psychological states (such as anxiety and depression) have been confirmed to promote the degradation of articular cartilage through mechanisms such as the activation of inflammatory factors and neuro-endocrine imbalance (20–22). The “Social Determinants of Health” framework proposed by the WHO also emphasizes understanding the mechanism of chronic diseases from a structural dimension. Our results provide empirical support for its application in secondary GJO.

Although the lifestyle score in this study showed a positive trend with the risk of secondary GJO, it did not reach statistical significance (*P* = 0.368). This may be attributed to: (1) high collinearity and mutual adjustment effects among lifestyle behaviors; (2) self-reported data collection, which may lead to information bias; (3) some lifestyle behaviors may have a weaker impact on non-weight-bearing OA. However, previous literature suggests that factors such as smoking, poor diet, and sleep disorders can be involved in the pathogenesis of secondary GJO through inflammatory pathways and chondrocyte apoptosis mechanisms (14, 23, 24). Therefore, further exploration of their temporal and mechanistic associations with GJO is still warranted in future studies.

We found a significant association between the metabolic factor score and the risk of secondary GJO (AHRs = 1.62). These metabolic disorders are often accompanied by systemic low-grade inflammation, dysregulation of adipokine secretion, and changes in the joint microenvironment, which in turn promote the destruction of cartilage matrix and bone remodeling (25–28). Notably, although secondary GJO is a non-typical weight-bearing joint OA type, it is highly correlated with metabolic risks, suggesting that its pathogenesis may transcend the traditional “mechanical OA” and “metabolic OA (MetOA)” paradigms.

Subgroup analysis showed that the predictive value of the SLM score for secondary GJO was more significant in women, men, and those under 60 years old. This finding suggests that the pathogenic effects of social, behavioral, and metabolic factors on secondary GJO are not limited to a specific gender group but have strong universality and generalizability. Specifically, the female population has physiological specificities in hormone levels (such as decreased estrogen), muscle mass, bone density, and pain sensitivity, which may make them more susceptible to the combined effects of social stress and metabolic burden, thereby accelerating the degenerative changes of the shoulder joint (3, 9, 29–31). Meanwhile, although men have traditionally been considered to have a lower incidence of OA, they are more commonly affected by metabolic abnormalities (such as abdominal obesity and lipid disorders), which may influence shoulder joint structure through adipokine imbalance and chronic inflammation pathways, explaining the significant association between SLM scores and risk. Additionally, the predictive role of SLM scores is particularly prominent in individuals under 60 years old, possibly because this group has not been widely exposed to age-related high-risk factors for OA (such as joint degeneration and endocrine changes), making the driving effects of social, lifestyle, and metabolic factors on disease development more sensitive and independent. This finding also emphasizes the concept of an “intervention window period,” suggesting that by identifying individuals with high SLM scores among young and middle-aged people and implementing targeted interventions, the onset of Secondary GJO could be significantly delayed, which holds significant public health value and implications for precision prevention.

The strengths of this study lie in the use of a large-scale population-based prospective cohort with comprehensive baseline measurements and long-term follow-up. Secondly, it proposed and validated the multi-dimensional integrated score (SLM score), which outperforms traditional single-factor approaches. Thirdly, it was the first to introduce an integrated assessment of social determinants and lifestyle indicators in the field of Secondary GJO, enriching the epidemiological model. Finally, it identified that the SLM score has a stronger discriminatory ability for specific populations (was present in both genders, and those under 60 years old), providing evidence support for individualized OA prevention strategies. However, it is necessary to acknowledge some limitations. Firstly, the diagnosis of Secondary GJO was based on hospital records, which may only cover moderate to severe cases and may underestimate milder or outpatient-treated cases. Secondly, some indicators were derived from self-reports, which may introduce information bias. Thirdly, the participants in this cohort were mainly of white ethnicity and the recruitment age was limited to 40–73 years old, which may limit the applicability of the study to other ethnic groups and younger populations. Fourthly, the SLM score was calculated using an equal-weighted sum, without considering the differences in risk contributions of each factor. Future studies may consider optimizing the model through regression weights or machine learning methods. Additionally, this study did not include some specific risk factors for Secondary GJO, such as occupational shoulder load intensity, genetic susceptibility, and history of sports injuries, which need to be further supplemented and verified in subsequent studies. Finally, although this study was based on a prospective design, due to its observational nature, the results can only reveal associations rather than causal relationships. Future studies still need to rely on longitudinal intervention studies or mechanistic studies to clarify the causal pathways.

## 5 Conclusion

This study found a significant association between a higher SLM score at baseline and an increased risk of Secondary GJO at the end of follow-up. Among them, social determinants and metabolic factors have a particularly prominent impact on Secondary GJO, suggesting that modifiable social determinants and metabolic factors have significant intervention potential in the primary prevention of Secondary GJO. Compared to uncontrollable factors such as age and gender, the SLM score provides a practical quantitative tool for clinical identification of high-risk populations for Secondary GJO, which is conducive to promoting evidence-based individualized intervention strategies.

## Data Availability

The raw data supporting the conclusions of this article will be made available by the authors, without undue reservation.
