# Supplementary material for: The association between social determinants, lifestyle and metabolic factors and the onset of secondary glenohumeral joint osteoarthritis: a cohort study of adults in the UK
Source: Front Public Health. 2026 Jan 27;13:1718963. doi: 10.3389/fpubh.2025.1718963 (PMC12888868; doi:10.3389/fpubh.2025.1718963)
Supplement: Supplementary file 1 [file Supplementary_file_1.docx]

**Supplemental information**

**Table-S1**. Construction of SLM Score and Summary of Risk Factors

**Table-S2**. Construction of the simplified Mediterranean diet score (aMED simplified version)

**Table-S3**. Identifying cases of Secondary GJO based on ICD10

**Table-S4**. The AUC, cutoff values, sensitivity, and specificity of ROC curve analysis.

**Table-S5**. The relationship between the SLM score at baseline and the risk of Secondary GJO at the end of follow-up

**Figure-S1**. The relationship between individual scores of social determinants, lifestyle and metabolic factors at baseline and the incidence of Secondary GJO at the end of follow-up

**Figure-S2**. The RCS curves of individual scores of social determinants, lifestyle and metabolic factors at baseline and the incidence of Secondary GJO at the end of follow-up

**Figure-S3**. The RCS curves of the SLM score at baseline and the incidence of Secondary GJO at the end of follow-up under the grouping based on age and gender

**Table-S1**. Construction of SLM Score and Summary of Risk Factors

| **Risk Factor** | **Measurement and definition** | **Reference category**  **Assignment** |
| --- | --- | --- |
| ***Social determinants* (0-4, scores)** | | |
| Education | Educational attainment was self-reported by individuals and classified into two groups: below high school and high school or above. | Low educational attainment--1 scores  High educational attainment--0 scores |
| Income | Household income was self-reported by individuals and is divided into three groups: low income, middle income, and high income. | low income--1 scores  middle/ high income--0 scores |
| Nature of work | Nature of work was self-reported by individuals and classified into two groups: involves heavy manual or physical, Yes or No | Yes--1 scores  No--0 scores |
| Emotion | Individuals seeking medical treatment for anxiety, tension or depression was self-reported by individuals and classified into two groups: Yes or No | Yes--1 scores  No--0 scores |
| ***Lifestyle factors*** **(0-5, scores)** | | |
| smoke | smoking status is self-reported by individuals and is divided into three groups: never smoked, used to smoke but quit, and currently smoke. | currently smoker--1 scores  never/former smoker--0 scores |
| drink | drink ing status is self-reported by individuals and is divided into three groups: never drink, used to drink but quit, and currently drink. | currently drinker--1 scores  never/former drinker--0 scores |
| aMED | For dietary patterns, we used the simplified Mediterranean diet score (aMED simplified version) and divided them into two groups: middle/high score and low score. | low score--1 scores  middle /high score--0 scores |
| Suggested Exercise Intensity | Whether the intensity of physical exercise meets the international recommendations and is divided into two groups: Yes or No | Yes--1 scores  No--0 scores |
| Quality of sleep | The quality of sleep is divided into two groups: Yes or No | Yes--1 scores  No--0 scores |
| ***Metabolic risk factors*** **(0-8, scores)** | | |
| Obesity （BMI） | Classifications are made in accordance with the World Health Organization (WHO) standards: normal (18.5 kg/m² ≤ BMI < 25 kg/m²), overweight (25 kg/m² ≤ BMI < 30 kg/m²), and obese (BMI ≥ 30 kg/m²). | Overweight/obese--1 scores  normal--0 scores |
| Central obesity (WHtR) | According to the standards of the European Congress on Obesity (CEO, 2019: http://ecoico2020.com/), classification is as follows: normal (0.4 - 0.49), borderline central obesity (0.5 - 0.59), and severe central obesity (≥ 0.6). | Borderline/severe central obesity--1 scores  normal--0 scores |
| Hypertension | Hypertension is defined as a systolic blood pressure greater than 140 mmHg or a diastolic blood pressure greater than 90 mmHg or an ICD-10 diagnosis, and is divided into two groups: Yes or No | Yes--1 scores  No--0 scores |
| Diabetes | Dyslipidemia is defined as hypertriglyceridemia (serum triglycerides greater than 1.7 mmol/L) or hypercholesterolemia (serum total cholesterol greater than 200 mg/dL) or ICD-10 diagnosis, and is divided into two groups: Yes or No | Yes--1 scores  No--0 scores |
| TG | Refer to the NCEP ATP III (Adult Treatment Panel III) standards of the United States, TG ≥ 1.7 mmol/L（150 mg/dL） | Yes--1 scores  No--0 scores |
| TC | Refer to the NCEP ATP III (Adult Treatment Panel III) standards of the United States, TC ≥ 5.2 mmol/L（200 mg/dL） | Yes--1 scores  No--0 scores |
| HDL | Refer to the NCEP ATP III (Adult Treatment Panel III) standards of the United States, HDL-C < 1.0 mmol/L（40 mg/dL） | Yes--1 scores  No--0 scores |
| LDL | Refer to the NCEP ATP III (Adult Treatment Panel III) standards of the United States, LDL-C ≥ 3.4 mmol/L（130 mg/dL） | Yes--1 scores  No--0 scores |

**Table-S2**. Construction of the simplified Mediterranean diet score (aMED simplified version)

| **Core dietary components** | **Measurement and definition** | **Reference category**  **Assignment** |
| --- | --- | --- |
| ***aMED*** **(0-7, scores)** | | |
| Vegetable intake | Including all non-starchy vegetables (excluding potatoes) | >=once a day or more --1 scores |
| Fruit intake | Including fresh fruits or dried fruits, but excluding fruit juices. | >=once a day or more --1 scores |
| Grain/Whole grain intake | Such as brown rice, whole wheat bread, oats, millet, buckwheat, etc. | >=once a day or more --1 scores |
| Fish intake | Including marine fish, freshwater fish and shellfish, etc. | >= twice a week --1 scores |
| Legume intake | Including soybeans, black beans, kidney beans, chickpeas, and bean products (such as tofu), etc. | >= twice a week --1 scores |
| Nuts intake | Including walnuts, almonds, cashews, hazelnuts, peanuts (un-fried), etc. | >= 3 times a week --1 scores |
| Red meat and processed meat intake | Including red meats such as pork, beef and mutton, as well as processed meat products. | < twice a week --1 scores |
| >= 3 scores--middle /high score; <3 scores--low score | | |

**Table-S3**. Identifying cases of Secondary GJO based on ICD10

| **Diagnosis** | **ICD codes** | **Details** |
| --- | --- | --- |
| Secondary GJO | M19.11 | Post-traumatic arthrosis of other joints (Shoulder region) |
|  | M19.21 | Other secondary arthrosis (Shoulder region) |

**Table-S4**. The AUC, cutoff values, sensitivity, and specificity of ROC curve analysis.

|  | AUC | cutoff values | sensitivity | specificity |
| --- | --- | --- | --- | --- |
| Social determinants | 0.621 | 0.0032 | 0.75 | 0.46 |
| Lifestyle factors | 0.591 | 0.0035 | 0.634 | 0.527 |
| Metabolic risk factors | 0.585 | 0.00369 | 0.564 | 0.585 |
| SLM scores | 0.778 | 0.006052 | 0.5842 | 0.8337 |

**Table-S5**. The relationship between the SLM score at baseline and the risk of Secondary GJO at the end of follow-up

| SLM (0-14, scores) | Model 1 | *P* value | Model 2 | *P* value | Model 3 | *P* value |
| --- | --- | --- | --- | --- | --- | --- |
| Low scores | ref |  | ref |  | ref |  |
| Middle scores | 2.19 (1.28-3.77) | **0.004** | 2.15 (1.25-3.70) | **0.006** | 2.15 (1.25-3.70) | **0.006** |
| High scores | 3.84 (1.92-7.69) | **0.0001** | 3.74 (1.87-7.51) | **0.0002** | 3.75 (1.87-7.51) | **0.0002** |
| Model 1: Adjust No  Model 2: Adjust Age.  Model 3: Adjust Age、Sex、Race. | | | | | | |

**Figure-S1**. The relationship between individual scores of social determinants, lifestyle and metabolic factors at baseline and the incidence of Secondary GJO at the end of follow-up


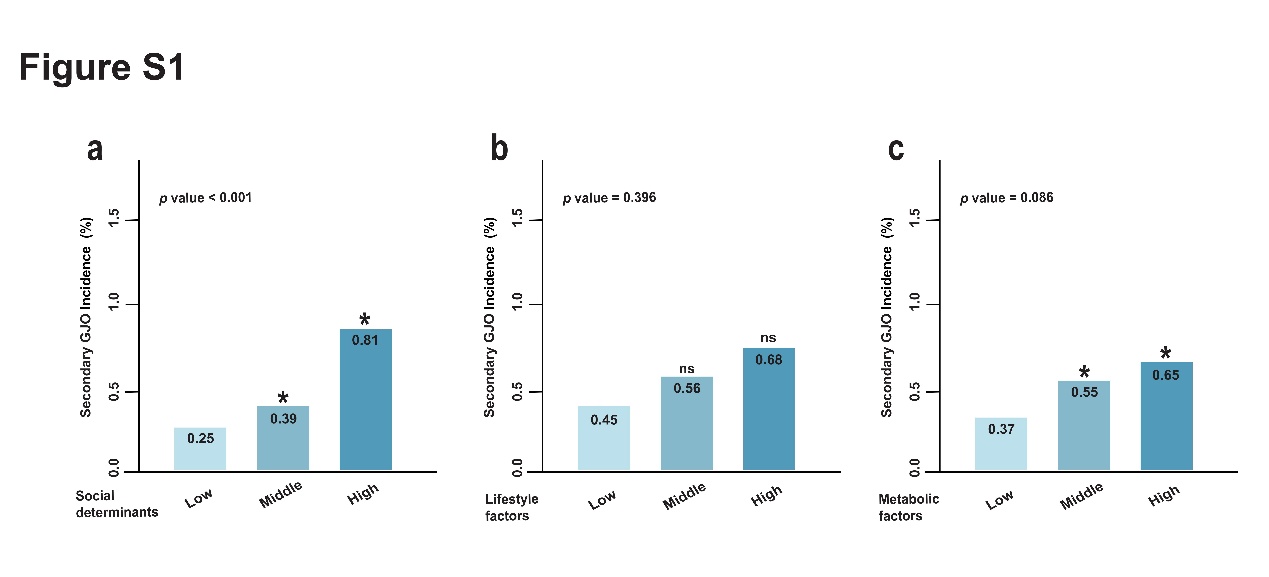


**Figure Legend:** Bar graphs show the incidence of Secondary GJO (%) across low, middle, and high levels of,

(a) Higher scores in social determinants were significantly associated with an increased incidence of secondary GJO (*P* < 0.001);

(b) Lifestyle factors showed a non-significant association with secondary GJO incidence across score groups (*P* = 0.396);

(c) Metabolic factors demonstrated a borderline association with secondary GJO incidence (*P* = 0.086), with both middle and high groups showing a modest increase compared to the low group.
**P* < 0.05 compared with the low group; ns, not significant.

**Figure-S2**. The RCS curves of individual scores of social determinants, lifestyle and metabolic factors at baseline and the incidence of Secondary GJO at the end of follow-up


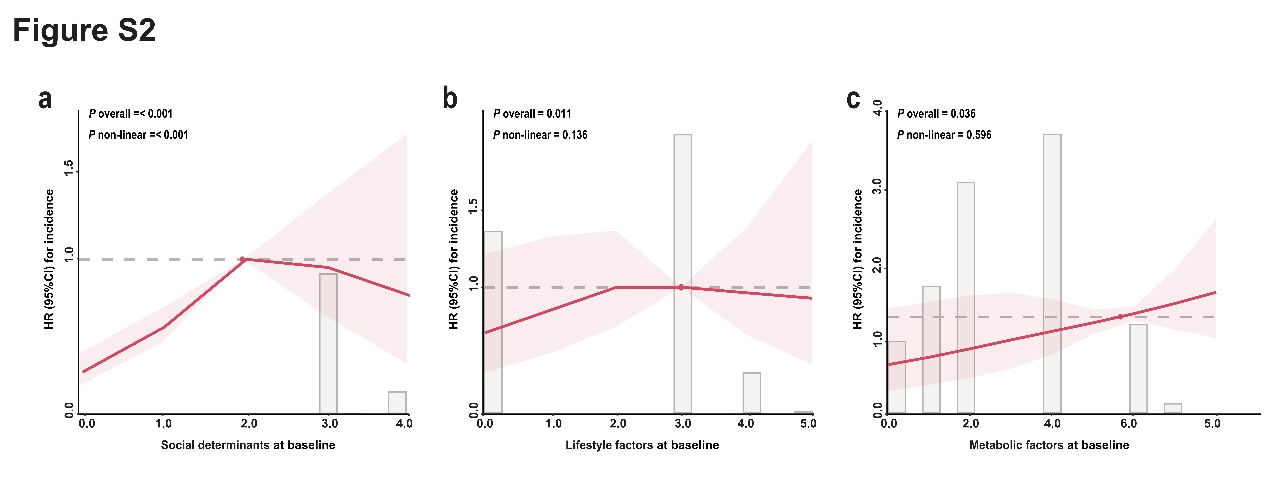


**Figure Legend:** Dose-response relationships between social determinants, lifestyle factors, and metabolic factors at baseline and the incidence of secondary GJO. Restricted cubic spline (RCS) models were used to explore the associations between each component score and the risk of secondary GJO.

(a) A significant non-linear relationship was observed between social determinant scores and the risk of secondary GJO (*P* overall < 0.001, *P* non-linear < 0.001).

(b) No significant non-linear association was detected between lifestyle factor scores and secondary GJO risk (*P* overall = 0.011, *P* non-linear = 0.135).

(c) A linear association was found between metabolic factor scores and secondary GJO risk (*P* overall = 0.036, *P* non-linear = 0.596).

Solid red lines represent hazard ratios (HRs) with shaded areas indicating 95% confidence intervals. Vertical grey bars denote the distribution of participants across score values.

**Figure-S3**. The RCS curves of the SLM score at baseline and the incidence of Secondary GJO at the end of follow-up under the grouping based on age and gender


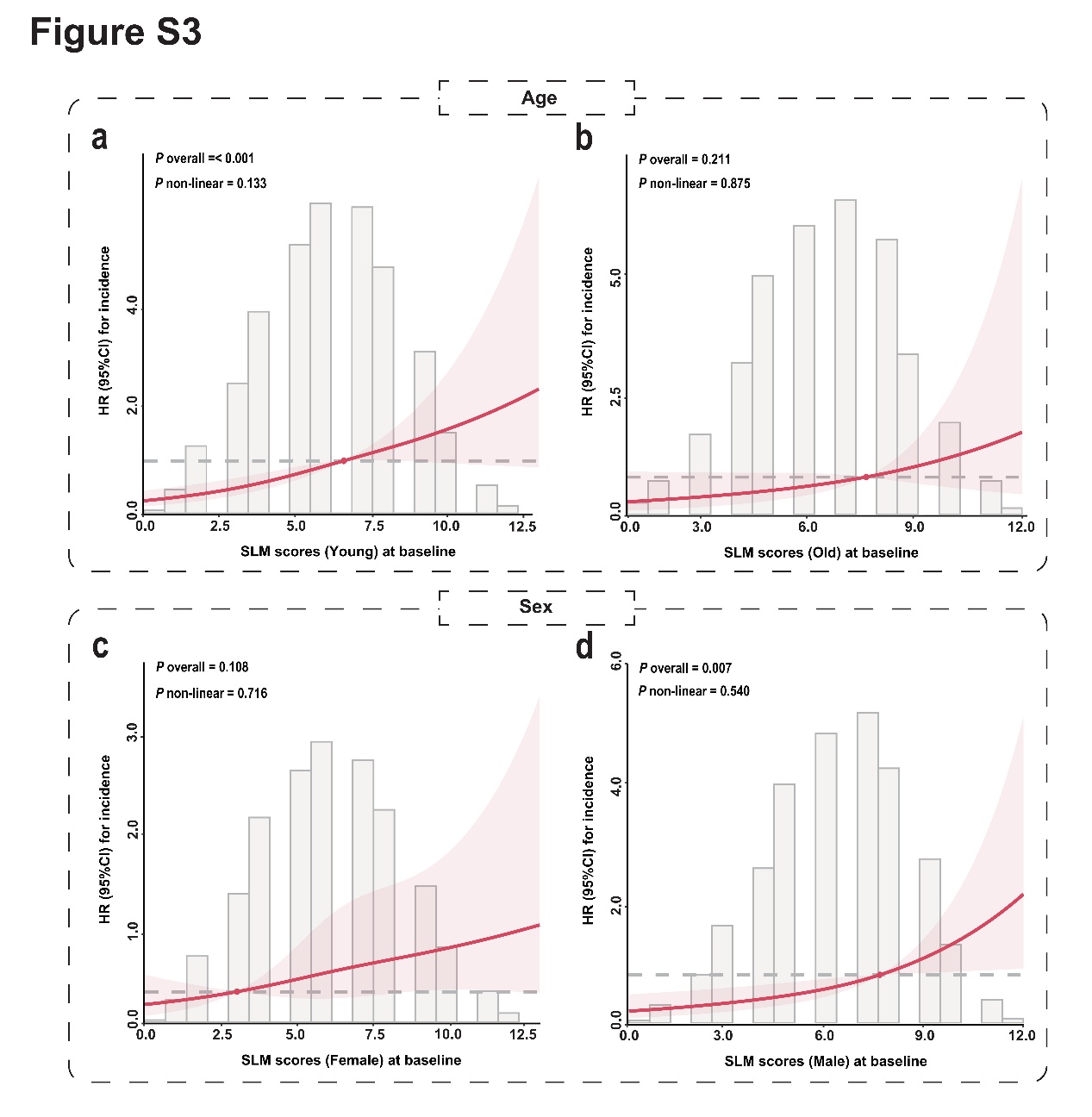


**Figure Legend:** Stratified dose-response relationships between baseline SLM scores and the risk of secondary GJO by age and sex.
RCS analyses were conducted to evaluate the association between SLM scores and secondary GJO incidence within different subgroups.
(a) Among younger participants (<60 years), a significant linear association was observed (*P* overall < 0.001, *P* non-linear = 0.133).
(b) In older participants (≥60 years), no significant association was found (*P* overall = 0.211, *P* non-linear = 0.875).
(c) Among females, the association was not statistically significant (*P* overall = 0.108, *P* non-linear = 0.716).
(d) Among males, a significant linear association was observed (*P* overall = 0.007, *P* non-linear = 0.540).

Solid red lines represent hazard ratios (HRs), and shaded areas represent 95% confidence intervals. Grey bars indicate the distribution of participants across the range of SLM scores.
